# Case report: Chronic pain in a pediatric patient with late-onset pompe disease

**DOI:** 10.3389/fpain.2023.1244609

**Published:** 2023-10-06

**Authors:** Amanda Cao, Raquel van Gool, Emma Golden, Benjamin Goodlett, Carlos Camelo, Simona Bujoreanu, Walla Al-Hertani, Jaymin Upadhyay

**Affiliations:** ^1^ Department of Anesthesiology, Critical Care and Pain Medicine, Boston Children’s Hospital, Harvard Medical School, Boston, MA, United States; ^2^ Division of Genetics and Genomics, Boston Children’s Hospital, Harvard Medical School, Boston, MA, United States; ^3^ Department of Psychiatry, McLean Hospital, Harvard Medical School, Boston, MA, United States

**Keywords:** pompe disease, lysosomal storage disease, pain, analgesia, depression, ADHD

## Abstract

Pompe disease (PD) is a rare inherited metabolic disorder of deficient or absent acid alpha-glucosidase (GAA), resulting in defective lysosomal glycogen catabolism. Muscle weakness, respiratory deficiency and gastrointestinal symptoms are commonly monitored in PD. However, pain and associated psychological symptoms are less focused upon. A pediatric patient with late-onset Pompe disease (LOPD) comorbid with chronic pain is presented. Symptoms of pain in the feet were first reported between 6 and 7 years of age and were attributed to growing pains. Following progression of lower body pain, weakness, fatigue, and difficulties with ambulation, a thorough clinical assessment including genetic testing was performed, which led to a diagnosis of LOPD at 9 years of age. ERT with recombinant human alglucosidase alfa was subsequently started. The patient’s clinical status is compounded by depressed mood, anxiety, and attention deficit hyperactivity disorder, which may further exacerbate pain. A multidisciplinary pain treatment approach consisting of orthopedics, physical therapy, and psychosocial therapy aimed at enhancing pain coping skills is described for this LOPD patient. This case highlights the need for a greater understanding of pain generation and identification of optimized pain treatment approaches in children with LOPD that can be implemented alongside ERT.

## 1. Introduction

Pompe disease results from an autosomal recessive mutation in the acid alpha glucosidase (GAA) gene (1). This mutation yields deficient or absent GAA, which results in glycogen accumulation, particularly in skeletal, cardiac, and smooth muscles, but also within the central nervous system (2–5). Patients with late-onset Pompe disease (LOPD) present with symptoms after the first year of life, typically demonstrating weakness in the limb-girdle, lower extremity, and trunk muscles (6). LOPD patients are often treated with enzyme-replacement therapy (ERT), exerting beneficial effects on the clinical course and survival (7–9). However, patients may still experience a range of physical and centrally based symptoms (10, 11). We present a patient with LOPD and a history of pain that initiated during early childhood.

## 2. Materials and methods

### 2.1. Editorial policies and ethical considerations

This investigation was approved by the BCH Institutional Review Board (IRB). Informed consent was provided by the patient’s legal guardian and patient.

### 2.2. Clinical history

**Figure 1:**
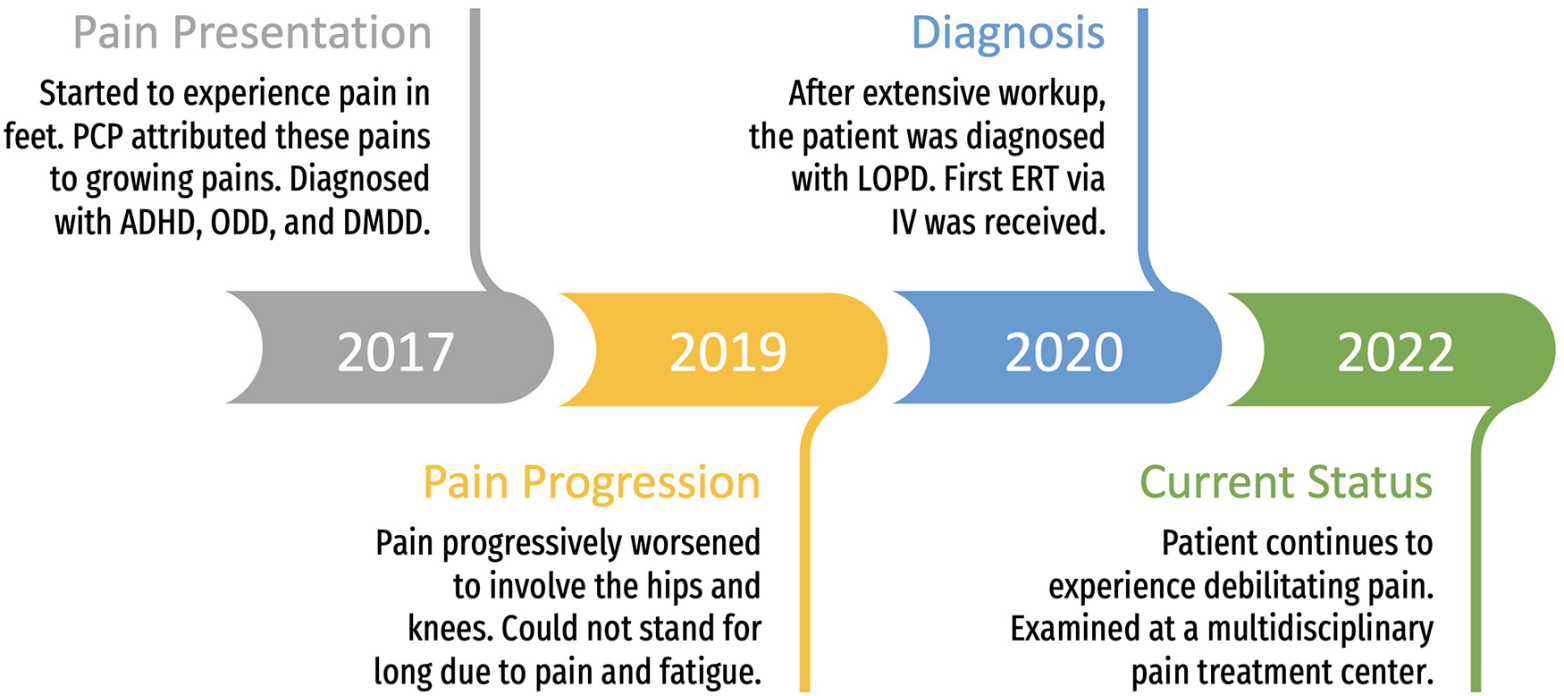
Clinical history and timeline.

A pediatric patient diagnosed with LOPD presented with bilateral, lower body pain (Figure 1). Pain was first noted at approximately 6 years of age and localized to their feet. Pain was considered a result of normal growing pains by the patient’s primary care provider. During this period, the patient also demonstrated signs of disruptive behavior (diagnoses of attention deficit hyperactivity disorder/ADHD, Oppositional Defiant Disorder/ODD, and Disruptive Mood Dysregulation Disorder/DMDD) in school and was prescribed Dexmethylphenidate, Risperidone for behavior and mood regulation, and Clonidine for sleep. The patient’s pain progressively worsened over 2 years, later affecting the bilateral anterior pelvis, knees, and feet, resulting in inability to stand for extended periods of time on hard surfaces or take part in physical activity (i.e., currently, needs to rest after standing on their feet for 1 h). After extensive clinical workup and genetic testing, a diagnosis of LOPD was confirmed at 9 years of age. Radiographic examination and musculoskeletal magnetic resonance imaging (MRI) of the lumbar spine and lower limbs were unremarkable for any bony lesions, joint abnormalities, nerve impingements or soft tissue lesions. Neuroimaging was also unremarkable.

Laboratory tests showed elevated creatine kinase (CK; 1558 units/L, Normal Range: < 177 units/L), Hexose tetrasaccharide (Hex4; 14.5 mmol/mol creatinine, Normal Range: <4 mmol/mol creatinine), and Alpha Glucosidase (2.70 pmol/punch/h, Normal Range: 10.88 pmol/punch/hour). Through genetic testing, two heterozygous pathologic variants were identified (c.−32-13T > G (Intronic) and c.525del (p.Glu176Argfs*45) in *GAA*. ERT with recombinant human alglucosidase alfa (1400 mg IV every two weeks, 20 mg/kg) was initiated shortly after the patient's LOPD diagnosis after significant symptom presentation affecting muscle strength and function, pulmonary function (coughing, wheezing, shortness of breath), and other patient reported outcomes (12). At the time of evaluation, the patient had been receiving ERT for 22 months.

## 3. Results

### 3.1. Assessment of pain

The patient’s mother sought additional clinical evaluation of the chronic pain. During a three-day examination period, the patient endorsed considerable pain, confirmed by the mother’s input, between the pelvis and feet, with pain severity experienced slightly more in the right compared to the left leg. The patient reported a pain level (0–10 scale) of 5 between hips and knees, a pain rating of 6 in the right ankle, and a pain rating of 9 in the right foot. Table 1 provides an overview of the presentation of pain [i.e., the Pain Frequency-Severity-Duration Scale (13)], as well as the psychological viewpoint of pain [i.e., the Pain Catastrophizing Scale and Fear of Pain Questionnaire (14, 15)]. The patient noted that pain is exacerbated when walking, standing, or sitting upright for prolonged periods (e.g., several minutes to an hour), but subsides with rest. Pain within both ankles and feet was described as aching, sharp, stabbing, and throbbing and at a level of 9. Pain localized to the bilateral anterior pelvis was described as sharp and ranged between 6 and 9. Presence of skin discoloration or tactile hyper- or hyposensitivity were absent in lumbar spine and lower limbs. However, mild bilateral trochanter and Achilles tendon tenderness was observed upon palpitation. Bilateral tightness of the Achilles tendon, bicep femoris, iliotibial band, and hip flexors was evident. At the time of the evaluation, pharmacological pain treatment included gabapentin (400 mg TID), baclofen (10 mg OD), and amitriptyline (25 mg OD).

**Table 1:** Pain-related scores and item responses.

Outcome measure	Score or item response
Pain Frequency-Severity-Duration Scale (PFSD)	
About how many days in the past 2 weeks have you been in pain?	14 days
About how long have you had a pain problem?	6 years
Would you describe your pain as recurrent or continuous?	continuous
On the days that you have been in pain, what has been your usual level of pain (0–10)	7
On average, how many hours has this usual pain lasted?	12–18 h
On the days that you have been in pain, what has been your worst level of pain (0–10)	9
On average, how many hours has this worst pain lasted?	4 h
Pain Catastrophizing Scale (PCS)	47: score is above 26, indicating high levels of catastrophic thinking
Fear of Pain Questionnaire (FOPQ)	76: score falls within range of 51–96, indicating a high fear of pain

Pain along with fatigue and muscle weakness, and the need to utilize assistive devices for ambulation has frequently limited or prohibited the patient from taking part in everyday physical activities at home or with peers at school—an aspect of the condition which has historically led to feelings of distress, embarrassment, irritability, social isolation, and low self-esteem. Additionally, the patient noted that physical symptoms were often elevated just prior to the bi-weekly ERT infusions.

### 3.2. Motor functioning

**Figure 2:**
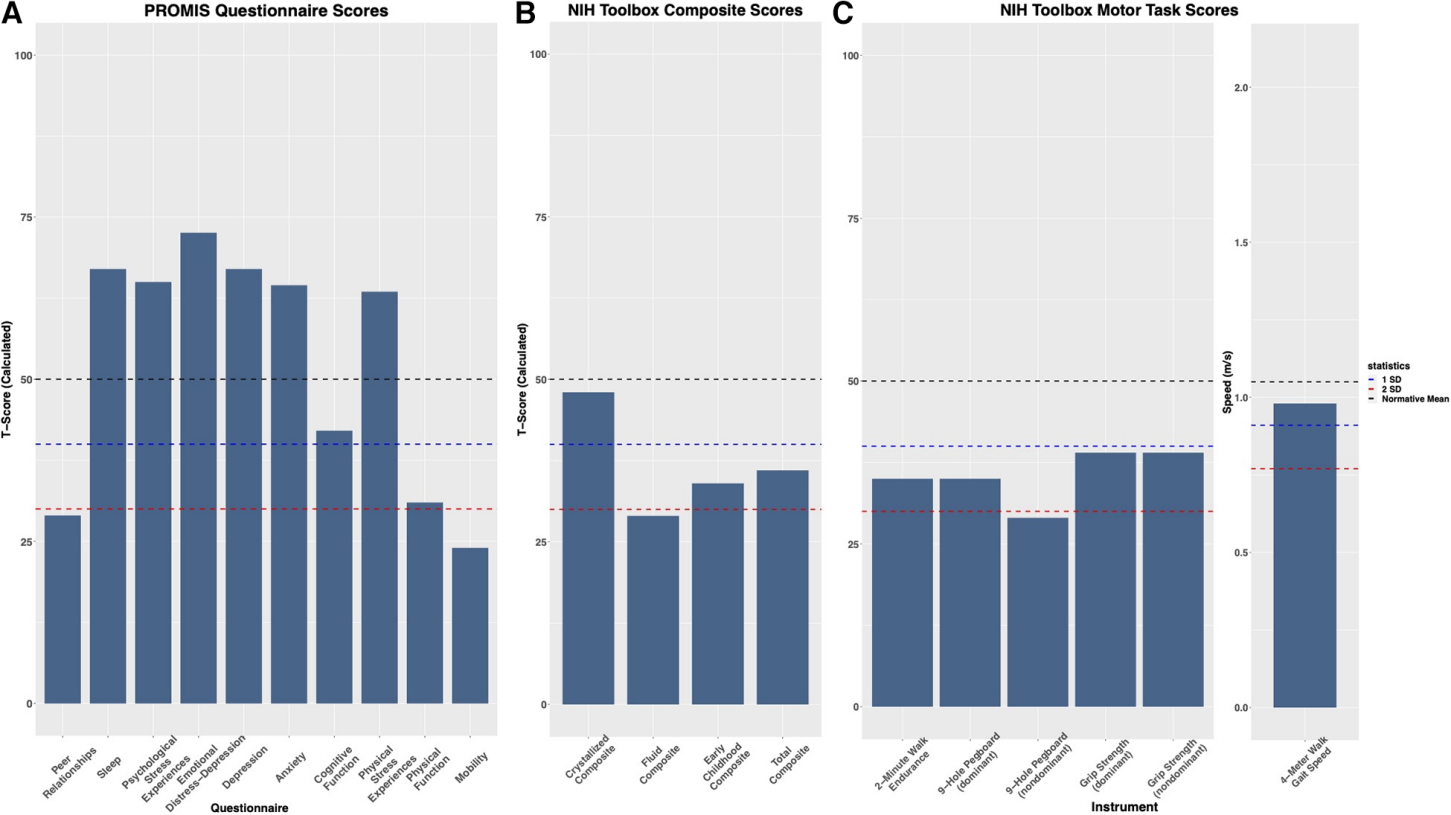
Evaluation of psychological and physical factors: (**A**) the patient shows higher level of depression, stress, anxiety, and sleep disturbances, and reduced quality of peer relationships, physical functioning, cognitive functioning, and mobility compared to the general population when assessed by patient-reported outcomes measurement information system (PROMIS) questionnaires. (**B**) On the NIH Toolbox Cognition Battery, the Total Composite Score and Early Childhood Composite scores factor in the Fluid Composite and Crystalized Composite Score to assess general cognitive function. For NIH Toolbox, T scores were corrected for gender, age, ethnicity, and education level. (**C**) Assessment of motor functioning utilizing the NIH Toolbox Motor Battery, evaluating dominant and nondominant function where accessible. On the 4 m Walk Gait Test, the patient scored (0.98 m/s) slightly below the age and gender-corrected mean (1.05 m/s).

In addition to the pain profile for this patient, upper and lower extremity motor functioning were assessed using the Patient-Reported Outcome Measurements Information System (PROMIS) questionnaires and the National Institutes of Health (NIH) Toolbox Motor Battery, respectively (Figure 2). The patient scored lower than the normative mean by approximately 1 standard deviation (SD) on upper (9-Hole Pegboard Dexterity Test and grip strength tests) and lower (2 min Walk Endurance Test and 4 m Walk Gait Test) extremity tasks. During upper extremity assessments, the patient showed little to no fatigue. However, for the lower extremity tasks, during the 2 min Walk Endurance Test and the Standing Balance Test, both pain and fatigue were evoked.

### 3.3. Psychological and neurocognitive status

The patient presented to the multidisciplinary clinic evaluation with a history of ADHD, ODD, and DMDD from six years of age. At the time of this evaluation, the mother reported that the patient had outgrown the ODD and DMDD diagnoses and was only treated with medication for ADHD and sleep. Based on the evaluation, the patient received a diagnosis of major depressive disorder (MDD), Generalized Anxiety Disorder (GAD), and Psychological and Behavioral Factors Affecting the Medical Condition, in addition to the child’s prior diagnosis of ADHD. Results on the PROMIS questionnaires further demonstrated elevated levels of depression, stress, anxiety, and sleep disturbances, along with reduced quality of peer relationships and cognitive functioning (Figure 2). The Total Composite and Early Childhood Composite score on the NIH Toolbox Cognition Battery was approximately 1 SD below the normative mean. The Fluid Composite T score, informing on problem-solving, reasoning, and memory encoding, was more than 2 SD below the normative mean. Treatment for the patient’s psychological symptoms includes dexmethylphenidate XR (40 mg OD, on school days only), sertraline (25 mg OD), clonidine (0.2 mg OD) for sleep, and psychotherapy (at the time of the multidisciplinary clinic evaluation, the bi-monthly psychotherapy was on hold due to logistical and health-care access barriers).

Psychological functioning was conceptualized through a biopsychosocial lens, to account for multiple contributing factors including physical/biological, psychological, and social, as well as developmental age, all likely contributing to the patient’s experience of and reaction to pain. The patient endures a severe and chronic illness that frequently imposes functional limitations leads to impairment across all relevant areas of life (school, peers, extracurricular activities, self-care, sleep, and family relationships and dynamics). In addition, their neurocognitive profile of ADHD and associated cognitive findings are likely to make them more susceptible than peers to feeling overwhelmed in the face of complex information and processing associated with the pain experience, pain management, and living with a chronic and potentially life limiting illness. Developmentally, this patient is at an age where the impact of medical issues can impact the sense of being a strong and healthy youth and further take a toll emotionally and socially by interfering with the achievement of developmentally appropriate milestones. Also, in relation to the young age, this patient presented with passive coping style (relying on distraction or asking for mother to do things for them), which becomes an additional vulnerability for emotional struggles in association with a lack of control over their own wellbeing.

## 4. Pain treatment approach

The patient’s pain in its current form is considered multifactorial, and thus a multidisciplinary pain treatment approach has been recommended. A rehabilitative pain treatment which combines physical and psychological therapies and focuses on improving function (as opposed to eliminating pain) was considered optimal for this patient (16). The biopsychosocial or functional-restoration approach entails a re-training of the nervous system to subside afferent drive or lower pain, and equally important, provide the patient with behavioral tools and strategies to enhance the mind-body interface, learn coping skills, regulate emotional and behavioral regulation, and improve communication with care providers. Learning strategies such as pacing activities are important parts of this process and can be incorporated as part of physical therapy treatment. A Cognitive Behavioral Therapy (CBT) strategy inclusive of deep breathing, guided imagery, and stress reduction was also recommended (17, 18). In parallel, considering biomechanical factors (i.e., tightness in the lower extremities), orthotic support for the feet and physical therapy involving aquatic therapy, home-based exercise, and a stretching routine with gradual pacing were indicated. No changes in the patient’s pharmacological treatment regimen were recommended.

## 5. Discussion

Herein, a pediatric patient with LOPD and severe persistent pain as well as cognitive and physical difficulties is described. Upon receiving a LOPD diagnosis and being placed on ERT, improvements in overall clinical status and physical functioning were noted, yet the patient’s pain as well as fatigue continue despite utilization of pharmacological (analgesics). Specifically, symptoms of pain as well as physical disability have greatly reduced quality of life, negatively impacting the child’s experience and performance at school, and introducing challenges in terms of taking part in social activities or developing social interactions. Further complicating the patient’s overall clinical status is a diagnosis of depression and anxiety, which may stem from living with a severe chronic illness, psychosocial limitations, presence of persistent pain, or environmental factors. While any conclusions on any causal interactions among pain and depression or anxiety are difficult to make, we propose that the current patient and likely others that present with a similar phenotype are prone to a cyclical and persistent allosteric load that can involve maladaptive neurobiological and physiological processes (19–22). Moreover, although the current patient’s pain-related symptoms and physical exam findings are not specific to LOPD, this case highlights the importance of early work up when presentation of pain or pain interference occurs alongside other signs and symptoms such as progressive muscle weakness.

Notably, the patient received an ADHD diagnosis at a young age and near the time that he first reported pain, which was initially solely attributed to growing pains. We speculate that a child’s early reports of pain likely warranted careful focus, specifically in relation to difficulties with attention or psychological presentation in general. Chronic pain has an interruptive effect on executive functioning, which, in school-age children, is frequently manifested in academic settings (23, 24). As such, pediatric pain may, in some instances, be the hidden cause behind behavioral and academic difficulties, specifically those attributed to problems with attention. Correct diagnosis and early intervention are important, more so in rare diseases and particularly when symptoms present during childhood development. Furthermore, Barr and colleagues (25, 26) hypothesized that early exposure to pain may redirect neurodevelopment in a way that renders an individual vulnerable to pain. This may likely contribute to one’s perception of pain in ways similar to that of the patient described here; mainly, high levels of pain catastrophizing and an increased fear of pain. Given the rarity of LOPD, accurate diagnoses of this illness may be difficult in the primary care setting and treating related symptoms. Additionally, providing incorrect diagnoses or inaccurate causes of symptoms likely further delay specialist evaluation. Significant diagnostic delays during childhood may be detrimental for disease progression but also increase the chances of developing physical and psychosocial symptoms that could potentially be mitigated with early and optimize treatment intervention.

As with many rare diseases, however, the focus of therapeutic interventions is rightfully placed on cardinal features of the disease. However, comorbid conditions such as chronic pain or neuropsychiatric manifestations may in some cases receive less attention despite their interference with physical or psychosocial health. Although longitudinal progress or treatment outcomes are unavailable at this time for this patient, we hope to underline in this investigative case the complexities regarding the spectrum of pain symptoms patients with Pompe disease may experience as well as the urgency for more focused attention to mental and physical health issues that may worsen the patients' experience with pain. Finally, while an immature neurological system in children with LOPD may on one hand be vulnerable to pathobiological processes either because of direct insult or a downstream effect, the neurodevelopmental status may in some cases, represent a therapeutic window for preventing or reversing maladaptive process that underpin conditions such as chronic pain.

## Data Availability

The raw data supporting the conclusions of this article will be made available by the authors, without undue reservation.
